# Engaging Patients with Late-Stage Non-Small Cell Lung Cancer in Shared Decision Making about Treatment

**DOI:** 10.3390/jpm11100998

**Published:** 2021-10-01

**Authors:** Ronald E. Myers, Shailesh M. Advani, Pamela Myers, Preethi Selvan, Gregory Garber, Brooke Worster, Neal Flomenberg, Andrew Chapman, Ralph Zinner

**Affiliations:** 1 Division of Population Science, Department of Medical Oncology, Sidney Kimmel Cancer Center, Thomas Jefferson University, Philadelphia, PA 19107, USA; Pamela.Myers@Jefferson.edu (P.M.); Preethi.Selvan@Jefferson.edu (P.S.); 2 Data Scientist, Terasaki Institute of Biomedical Innovation, 1018 Westwood Blvd, Los Angeles, CA 90024, USA; shailesh.advani735@gmail.com; 3 Department of Medical Oncology, Sidney Kimmel Cancer Center, Thomas Jefferson University Hospital, Philadelphia, PA 19107, USA; Gregory.Garber@jefferson.edu (G.G.); Brooke.Worster@jefferson.edu (B.W.); Neal.Flomenberg@jefferson.edu (N.F.); Andrew.Chapman@jefferson.edu (A.C.); 4 Department of Medical Oncology, Markey Cancer Center, University of Kentucky, Lexington, KY 40536, USA; Ralph.Zinner@uky.edu

**Keywords:** lung cancer, shared-decision making, treatment decision, non-small cell lung cancer, NSCLC

## Abstract

Few treatment decision support interventions (DSIs) are available to engage patients diagnosed with late-stage non-small cell lung cancer (NSCLC) in treatment shared decision making (SDM). We designed a novel DSI that includes care plan cards and a companion patient preference clarification tool to assist in shared decision making. The cards answer common patient questions about treatment options (chemotherapy, chemotherapy plus immunotherapy, targeted therapy, immunotherapy, clinical trial participation, and supportive care). The form elicits patient treatment preference. We then conducted interviews with clinicians and patients to obtain feedback on the DSI. We also trained oncology nurse educators to implement the prototype. Finally, we pilot tested the DSI among five patients with NSCLC at the beginning of an office visit scheduled to discuss treatment with an oncologist. Analyses of pilot study baseline and exit survey data showed that DSI use was associated with increased patient awareness of the alternatives’ treatment options and benefits/risks. In contrast, patient concern about treatment costs and uncertainty in treatment decision making decreased. All patients expressed a treatment preference. Future randomized controlled trials are needed to assess DSI implementation feasibility and efficacy in clinical care.

## 1. Introduction

According to 2021 Cancer Statistics, lung cancer will account for 235,760 new cancer cases and 131,880 cancer related deaths in the United States. The overwhelming majority (84%) of new lung cancer patients are diagnosed with non-small cell lung cancer (NSCLC). Of those cases, 57% present with late-stage disease [1]. There are a number of treatment options for patients with late-stage NSCLC, the mainstays of which are chemotherapy, targeted treatment, and immunotherapy, palliative radiation and palliative surgery and supportive care, and clinical trial participation. Clinicians are trained to identify a personalized treatment strategy based on a many factors, including patient age, health status, comorbidity profile, clinical presentation of disease, molecular and tumor characteristics, and patient preference [2,3,4,5,6,7].

As part of determining a treatment plan, clinicians are encouraged to engage patients in shared decision making (SDM) about recommended options [8,9,10,11]. Treatment SDM is a patient-centered process in which the clinical team provides patients and caregivers with information about the diagnosis, prognosis, and available treatment options; elicits patient values related to recommended treatment alternatives; clarifies personal preference related to the options, and helps the patient choose an option that is consistent with personal goals and optimal clinical care [12]. Decision support interventions (DSIs), which have also been referred to as “decision aids”, can help to facilitate treatment SDM.

Historically, DSIs have been developed in educational booklets and videos designed to present information about health care decisions to be made [13]. DSIs have also taken the form of interactive computer-based or online software applications that provide patients with information about available health care alternatives and help patients clarify their preferences [14]. Exposure to such DSIs in health care has been shown to increase patient knowledge, reduce patient decisional conflict, and boost patient satisfaction with care [15,16]. It has also been reported that DSI use can increase satisfaction with physician-patient communication about treatment and ease patient anxiety about treatment decision-making among patients diagnosed with late-stage cancer [17].

Our current project aimed to (a) Perform a scoping review to identify DSIs developed for use with patients diagnosed with late-stage NSCLC, (b) Develop a DSI that provides patients with information about current treatment alternatives and elicits treatment preference, and (c) Assess DSI use in this patient population, and explore how DSIs can be integrated effectively in routine care.

## 2. Materials and Methods

**Figure 1:**
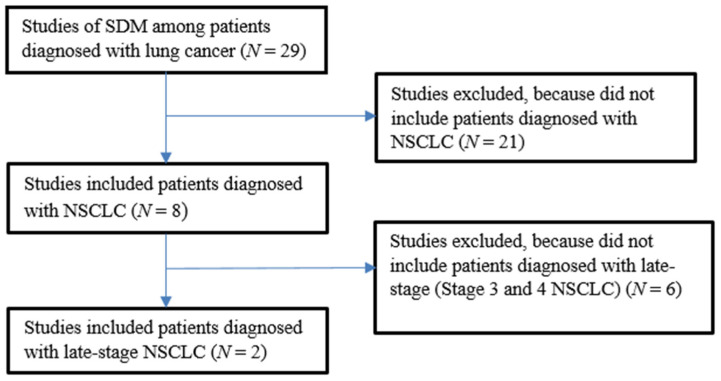
Studies of SDIs and SDM among Patients with non-small cell lung cancer (NSCLC).

Following Coulter et al., the research team searched the literature to identify studies of treatment SDM among patients diagnosed with lung cancer published from January 2000 to March 2020 [18]. This search involved searching databases: PubMed, Ovid (Medline), and Google Scholar. We included original research articles (observational studies and clinical trials) published in English. MeSH terms used to perform our search included: ‘shared decision making’, ‘lung cancer care’, ‘non-small cell’, ‘patient decision aid’, ‘sys-tematic review’, ‘meta-analysis’ and ‘development’, ‘evaluation’ were included in the search process. This effort initially yielded 29 publications that addressed SDM among patients diagnosed with lung cancer. Twenty-one reports did not focus on patients diagnosed with NSCLC. Full-text analysis of the remaining eight reports that included patients with NSCLC resulted in identifying two studies that evaluated DSI use among patients diagnosed with late-stage NSCLC [14,17]. Details of this process are presented in Figure 1.

### 2.1. Development of a DSI Prototype for Use with Patients Diagnosed with Late-Stage NSCLC

After completing the scoping review, the research team engaged a design panel to develop a DSI prototype that presented informational content related to current treatment options for patients diagnosed with late-stage NSCLC. We also engaged a physician panel and a patient panel to gain insights into provider and patient perceptions about the treatment SDM and utilization of the DSI prototype in practice.

### 2.2. Design Panel

This panel consisted of clinicians (two medical oncologists and two oncology nurses involved in caring for NSCLC patients) and three researchers. Based on iterative discussions about findings from the scoping review, design panel members decided it was essential to develop a new type of DSI. This new DSI would provide basic information about a range of treatment alternatives that answered patient questions, did not require patients to read a lengthy document on available options and view a video recording, and could be integrated into a clinical encounter. In addition, the panel agreed that it was essential to develop a DSI prototype as a template for presenting treatment information that could be modified to reflect emerging changes in treatment recommendations. Moreover, panel members decided that a tool should be developed to record patients’ preferences related to presented treatment options.

### 2.3. Physician Panel

Medical Oncologists from the Department of Medical Oncology at Thomas Jefferson University Hospital were recruited to participate in a semi-structured in-person interview on treatment shared decision making with patients diagnosed with late-stage NSCLC. In the interview, participants were asked to describe what treatment alternatives they were likely to recommend and how they helped patients make treatment decisions. In addition, the interviewer explained the DSI prototype and asked for feedback on the utility of the prototype and the feasibility of integrating the tool into clinical care.

All interviews were audio-recorded and transcribed. Interview transcripts from the physician panel were coded independently by two members of the research team (SS and PM) using NVivo 12 Pro (QSR International, Doncaster, Australia). Specifically, each research team member evaluated each interview segment to identify and code initial sub-themes, organize sub-themes into major themes, and ensure the major themes were unique. The PI of the project (RM) met with research team members to resolve any differences in sub-theme identification, coding, and organization.

### 2.4. Patient Panel

This panel included patients identified by the physician panel who were not enrolled in the DSI pilot study. Eligibility criteria included being diagnosed with late-stage NSCLC (either at baseline or recurring after surgery or radiation) and having had one of the following treatment regimens: chemotherapy alone, chemotherapy and immunotherapy, targeted therapy, or immunotherapy. Patients were excluded if they had comorbidity or psychological factors that would contraindicate participation in the study or if a patient was unable to provide informed consent or did not speak English.

For interested and eligible patients, in-person interview appointments were arranged. Patients received a gift card for $50 after the interview for their participation and time. The interviewer asked patients to describe their experience how they learned about their diagnosis, treatment possibilities, and prognosis. In addition, the interviewer asked patients to share what they learned and how they felt about their interactions with clinicians. Patients were also asked to review each care plan card and treatment preference report (which can be found in supplemental materials).

All interview transcripts from the patient panel were coded independently by two members of the research team (SS and PM) using NVivo 12 Pro (QSR International, Doncaster, Australia). The research team developed codes and identified significant themes based on a review of interview transcripts. As with the physician interviews, each research team member evaluated each interview segment to identify and code initial sub-themes, organize sub-themes into major themes, and ensure the major themes were unique. The PI of the project (RM) met with research team members to resolve any differences in sub-theme identification, coding, and organization.

### 2.5. The Pilot Study

The research team planned to engage clinicians from the physician panel in identifying and consenting patients for inclusion in a pilot study of the DSI prototype. Eligibility criteria included patients who were 18 or more years of age, diagnosed with advanced NSCLC (either at presentation or at first recurrence after surgery or radiation), could read and speak English, and were approved by their oncologist for contact. Once a patient was deemed eligible and was referred, a research assistant attempted to contact the patient by telephone to explain the study, confirm eligibility, and assess their willingness to participate. If the patient indicated an interest in the study, the next scheduled office visit was set to complete study procedures.

Before the scheduled visit, each participant’s oncologist informed the research team of the two major treatment options to be discussed at the visit. These treatment options were evaluated by a Thomas Jefferson University financial advocate, who obtained specific information about the patient’s current insurance plan, coinsurance, copay, and deductibles. Using this information, the financial advocate provided the research team with estimated participant out-of-pocket maximum costs related to the options. The research assistant entered this information into a personally tailored care plan card that described the treatment options recommended for each participant and print two cards for each participant for use by an oncology nurse in the decision counseling session. At the beginning of the office visit, a study research assistant met each participant, reviewed study procedures, obtain consent, and administer a baseline survey questionnaire.

The baseline survey included single items designed to elicit patient belief that treatment for late-stage NSCLC was likely to result in a cure, awareness that there was more than one recommended treatment option for late-stage NSCLC, and concern about treatment cost. Response options for each of these items were “Yes”, “No”, and “Unsure”. The survey also included four items from the Decisional Conflict Scale [19], a measure used to assess the respondent level of uncertainty related to the treatment of NSCLC. Scale questions were: Do you feel sure about the best choice for you? Do you know the benefits and risks of each option? Are you clear about which benefits and risks matter most to you? and Do you have enough support and advice to make a choice? Participants could choose either a “Yes” or “No” response to each question. Responses were summed, resulted in scores that could range from 0 (extremely high decisional conflict) to 4 (no decisional conflict). In this instance, a score of <3 indicates greater uncertainty about which treatment option is the best choice, while a score of >3 indicates greater certainty about the best treatment choice. Baseline survey items noted here were also included on an exit survey administered by the research assistant at the end of the office visit.

After the research assistant administered the baseline survey, an oncology nurse engage each participant in a 15–20-min decision counseling session. During this session, the nurse review the two care plan cards with the participant and record participant questions related to card content. Finally, the nurse elicited participant preference for each treatment option (Prefer, Unsure, Do Not Prefer) and enter this information into the preference report. One copy of the report was given to the patient, and another copy was placed on the participant’s medical chart for review with their physician. After completing the decision counseling session and before the physician-patient encounter, the research assistant meet the patient and administered an exit survey. The exit survey included the same single items to assess patient perceptions included in the baseline survey. The exit survey also included the items used to determine baseline decisional conflict related to treatment. Participants then went on the complete their physician-patient encounter. At 30 days, the research team completed a medical records review to determine participant treatment status.

## 3. Results

### 3.1. Scoping Review

In a study that involved 20 patients diagnosed with metastatic NSCLC, Leighl et al. reported on participants who had completed an oncology visit at Princess Margaret Hospital in Canada [10]. Participants were provided a 25-page booklet that included information about chemotherapy and available clinical trials, along with an audio-recording for use at home. Another study conducted at the Ottawa Regional Cancer Center in Canada by Fiset et al. involved 30 patients who were diagnosed with stage IV lung cancer and were provided information about chemotherapy treatment by an oncologist [20]. Following the clinic visit, a total of 20 patients agreed to review treatment information presented in a booklet and an audio-recording. In these studies, most patients reported that receiving treatment information in an informational booklet/audio-recording format was helpful in treatment decision making (75% and 100%, respectively) [20].

As shown in Supplementary Table S1, both studies showed that patient treatment knowledge increased significantly (*p* < 0.001) and patients reported favorably views of the interventions. The Fiset et al. study showed that decisional conflict decreased significantly (*p* < 0.001), and Leighl et al. reported that patient anxiety decreased (*p* = 0.04). Importantly, these studies involved patients who were already undergoing treatment or had previously discussed treatment with a clinician. In addition, the studies tested DSIs that provided information in a self-guided fashion on a limited array of treatment options; and the DSIs required patients to take action on their own to learn about treatment options. That is, they tested DSI effects outside the context of a treatment consultation visit, rather than testing the impact of providing information about treatment options as part of an oncology consultation. Moreover, they addressed a very limited number of treatment options. Given the changing increasing number of treatment alternatives available to patients with NSCLC, there is a need for DSIs that can be used to facilitate treatment shared decision making about current options [10,20].

### 3.2. Design Panel

The design panel developed a 1-page format called a “care plan card” to display treatment options commonly offered by oncologists. A care plan card was developed for each of six treatment options, including: chemotherapy, chemotherapy and immunotherapy, immunotherapy, targeted therapy, clinical trial participation, and supportive care. In addition, panel members identified a set of common questions patients asked about treatment and agreed on simple answers to each question. The cards included answers to the following questions: (1) What can I expect from the Treatment? (2) What agent(s) will I receive? (3) Will my symptoms be treated? (4) Will I be offered radiation or minor surgery if it could relieve my symptoms? (5) How often do I get Treatment? (6) How often will I need X-rays or CT scans? (7) How often will I need blood tests? (8) What are the side effects? (9) What is the out-of-pocket cost of Treatment? A generic response to questions 1–8 was included, along with an open-ended response to question 9 that was intended to be specific to each patient’s insurance coverage. The design panel also developed a 1-page report referred to as a treatment preference report to augment the care plan cards. This document allowed the patient to choose one of the following responses for each treatment option presented: Prefer, Undecided, and Do Not Prefer. Patient responses could be recorded on the report, along with any questions the patient may want to ask the physician during the ensuing physician-patient encounter. These tools are included as Supplementary Materials.

### 3.3. Physician Panel

Clinicians (*N* = 9) were recruited and interviewed. Four themes emerged from these interviews: (1) Tailoring treatment to the patient; (2) Physician-patient communication; (3) Engaging members of the clinical team, and (4) Integrating DSIs into clinical care.

In regard to the first theme, respondents highlighted the importance of tailoring treatment plans to an individual’s unique physical and clinical characteristics. For example, Physician 6 noted, “Some patients have other problems such as rheumatoid arthritis and ulcerative colitis, which can color the decision to use immunotherapy because the risk is a bit higher…so, you need to know other things about the patient to make the best decision”. Physician 8 also illustrated this point by saying, “For lower performance status, the patient may be more likely to develop toxicity from the therapy, and therefore, chemotherapy may pose more of a risk to that person. Therefore, the consideration for immunotherapy alone or supportive care should at least be considered and discussed…” Another respondent (Physician 3) highlighted the increasing importance of genetic test results in clinical decision making, “I think the genetic mutations are the things we’re looking for more and more these days. I think that’s—focuses more towards targeted therapies. The targeted therapies can be very helpful in the patient’s individual situation and result in some pretty dramatic responses with treatment”.

For the second theme, most physicians reported that establishing good physician-patient communication involves both providing information and asking about the patient’s treatment goals. As explained by Physician 2, “educating the patient, married with identifying patient goals, especially related to having an acceptable quality of life, is the way that a medical decision is arrived at, so that it is able to fit both sides of that equation”. Physician 6 also noted, “most patients would want to hear from their physician what the disease is, how the disease might be treated, whether or not they have a mutation and in general about the treatment”. Physician 9 noted, “I…talk to (patients) about the risks and benefits, what toxicity there is between the different options, and talk about what their preferences are, what their goals of therapy are”. Another physician (5) commented, “I think that’s important for patients when they’re having a difficult time deciding whether to take active treatment if they have a poor physical status or fairly significant tumor burden (then) having further knowledge of expected outcomes with the treatment can shape better expectations and help the decision for the patient”. Several physicians mentioned the need to address the role of family members, especially with patients for whom supportive care is recommended as an option to consider.

For the third theme, physicians acknowledged that nurses and other oncology team members could play an important role in SDM. As expressed by Physician 6, “…most patients would want to hear from their physician what the disease is, how the disease might be treated, whether or not they have a mutation and in general about the treatment”. However, an oncology nurse, a nurse practitioner, or social worker on the care team could help to address patient needs for additional information, “…if people are asking a little bit more about side effects or practical stuff like that, then I think it needs to be someone that has some knowledge about that…a person that had some additional education about those options…whether it’s a nurse, or a research coordinator, or a social worker”.

Lastly, in regards to the fourth theme, respondents thought that implementation of the DSI with patients could be useful after the physician decided on a treatment in order to increase patient understanding and adherence but were unsure about how/when the use of the tool could be integrated into clinical care. As noted by Physician 7, “…it (DSI use) shouldn’t be at the first (visit)…when the patient’s given the diagnosis. It should be after the diagnosis has been given (in) the evaluation period”. Physician 1 recommended, “Well, I think…the patient may wish to take it (results of the DSI session) home and think about it for some time. I think it may be helpful to have sort of a telephone follow-up…in expediting the next steps in treatment”.

### 3.4. Patient Panel

Ten patients were interviewed. The patient interview transcripts resulted in the identification of three major themes: (1) Patient and caregiver involvement, (2) Physician-patient communication, (3) Engaging members of the clinical team, and (4) Integrating DSIs into clinical care.

Regarding Patient Theme 1, patients commonly reported that a family member or friend accompanied them to their office visits, and they explained that these caregivers play an important role in helping them understand information imparted by the clinician. One patient (Patient 3) noted, “My husband was with me every step of the way”. Patient 4 said, “(The physician) laid out this—the original protocol, which is the one I’m doing. And then that was the one. We picked this one because I wanted to have a (good) quality of life”. In some cases, however, patients felt that the process of engaging caregivers in treatment decision making was suboptimal. As noted by Patient 7, “We (patient and caregiver) would be presented with the information from the provider. He allowed us to ask questions. And, the questions weren’t necessarily answered with the information that we needed to feel like we were empowered”.

For theme 2, patients commonly reported that physician-patient communication tended to focus on the diagnosis, test results, a recommended treatment approach, and deciding on whether or not to follow the recommendation. Patient 6 described the process as follows, “(The doctor) explains to the patient, okay, this is what you’re seeing, this is what we found out, and this is what all the tests—the conclusions to all the tests were. And now, together, we both need to make a decision”. As described by Patient 1, this process varies by the physician as, “Some physicians explain everything in words, verbally. Some physicians give printed materials to read. Different physicians do this differently”. Many patients pointed out that physicians need to use different communication methods to ensure patient understanding. In this regard, patient 6 explained that to be successful in communicating with patients, “The doctor (has) to understand a person’s level of intelligence…you (can’t) make it too technical, because a person might not understand it. You’d have to be able to make it where the person feels comfortable…”

For the third theme, patients viewed their interactions with the clinical team as quite important, especially those that took place with oncology nurses at the time of their office visit. Patient 3 explained, “…I tell you, you have to have a special nurse to be able to have the knowledge and maintain the relationship, doctor/patient, nurse/patient, two different things”. To optimize care, patient 3 highlighted the importance of the nursing staff as follows, “It’s gotta be the special nurse that has it in her heart to be able to relate and have empathy for the one who has cancer”. Patient 4 commented on the role of nurses on the clinical team as follows, “And having the nurses involved in helping to explain things that are going to happen. …she also knows the medicine and the pros and cons, because I think that’s what they get taught”.

Lastly, for the fourth theme, patients expressed that it was essential to making the tool available as part of the treatment decision making. In this regard, Patient 3 said that the use of a DSI would help to engage patients in the treatment decision making process, “Rather than just say this is what you have to do, we want you to be part of that process of understanding and deciding”. Regarding the option cards, another patient (Patient 6) said, “You should get this card when you initially meet with the doctor, so you will know what to expect. So, if you decide to change your mind…at least you know what you’re facing”. Another patient commented on the cards’ informational content, noting that treatment is intended to increase the length and quality of life. I think that’s good to get that right out in front and to explain the side effects”. Patient reactions to the summary report were also positive, as noted by Patient 6, “Because it’ll give the patient something to look at, visually…And it would allow your doctor to explain to the patient, (and) make his own decision, too”.

Major themes identified through interviews conducted with physician panel and patient panel participants are summarized in Table 1.

### 3.5. The Pilot Study

The research team worked with medical oncologists currently involved in treating late-stage NSCLC to identify and consent patients in a pilot study of the DSI prototype. *N* = 5 patients were recruited and completed all study tasks.

#### 3.5.1. Demographic Characteristics

Table 2 shows that among study participants, three were female, four were white, and one was African American. Two participants were married, and two had a high school education.

#### 3.5.2. Change in Perceptions about Treatment

Data presented in Table 3 shows that at baseline, two participants believed that treatment was likely to result in a cure, one did not think that a cure was possible, and two were unsure. Findings from the exit survey indicate that one of the participants who were uncertain about the likelihood of a cure reported that a cure was unlikely. At baseline, only two participants stated that they knew what treatment options were available to them. Still, at the time of the exit survey, all participants reported that they were aware of their treatment options. At baseline, three participants expressed concern about treatment cost, but only one said this concern on the exit survey.

#### 3.5.3. Change in Decisional Conflict about Treatment

Table 4 shows that at baseline, participants had a mean decisional conflict scale score of 2.2, which indicates uncertainty about which treatment option was preferred. On the exit survey, participants reported a mean decisional conflict score of 3.2, indicating that their level of decisional conflict was reduced, and they felt more certain about their preferred treatment choice.

#### 3.5.4. Recommended Treatment Options, Treatment Preference, and Treatment Status

As indicated in Table 5, each participant was offered a different combination of treatment options. The recommended treatment option was as follows: Participant 1—chemotherapy and immunotherapy or immunotherapy; Participant 2—chemotherapy and immunotherapy or chemotherapy; Participant 3—immunotherapy or a clinical trial; Participant 4—chemotherapy or a clinical trial; and Participant 5—immunotherapy or supportive care. Participant treatment preferences recorded after the nurse-guided decision counseling session were: Participant 1—chemotherapy and immunotherapy; Participant 2—chemotherapy and immunotherapy; Participant 3—immunotherapy; Participant 4—chemotherapy alone; and Participant 5—immunotherapy. At 30 days after the office visit, a review of medical records indicated that Participant 1 had not made a treatment decision; Participant 2 was undergoing chemotherapy and immunotherapy, Participant 3 enrolled in a clinical trial, and Participants 4 and 5 received chemotherapy.

## 4. Discussion

When we initiated this study, there were a small number of published reports on DSIs that had been developed for use with patients diagnosed with NSCLC. We discovered that existing DSIs focused on a small number of treatment options. In addition, the tools provided a substantial amount of complex information either in print form or online that answered a limited range of patient questions. In addition, published reports did not focus on the role clinical team members could play in preparing patients and family members for shared treatment decision-making. We sought to address these issues by developing a 1-page care plan option card, each of which provided generic answers concisely to questions asked by patients. The cards were tailored for individual patients by providing information on potential out-of-pocket costs related to care. This DSI allowed clinicians to select treatment options they planned to discuss with their patients and allowed patients to gain insight into the nature of those options before the physician-patient encounter. Oncology nurses also guided patients through a simple exercise to elicit patient treatment preference.

This DSI prototype differs from others reported in the [10,20] in several respects. First, the care plan option cards are designed to provide basic information about the range of treatment options available to patients. Second, basic information on the cards is generically presented and easy to understand, especially when the presentation is guided by a nurse or other oncology team member. Third, information on the card can be tailored to address specific patient concerns, such as treatment-related costs. Furthermore, this patient and family member education process is augmented by engaging oncology nurses in guiding patients through a brief preference clarification exercise, which can be made available immediately to patients and providers to determine a course of treatment that makes sense to patients and providers. In our view, this type of DSI is relatively simple, can be adapted for use in various practice settings, and is feasible for integration into the clinical workflow. Notably, the DSI also serves to engage the clinical team in the treatment shared decision-making process.

In the pilot study, we found that oncologists embraced the opportunity to identify care plan option cards for review by patients before an ensuing patient encounter; oncology nurses were able to review selected cards with patients and family members at the beginning of a clinic visit. Our study found that using the DSI prototype was feasible while the patient was waiting for their oncologist consultation. We also determined that deployment of the DSI prototype had beneficial effects on knowledge and perceptions related to treatment decision-making in a subset of patients included in the pilot study. The provision of simple care plan cards enabled patients to learn basic information about treatment alternatives from an oncology nurse on the care team before consulting with an oncologist.

The potential value of deploying such interactive DSIs for patients with advanced cancer is highlighted by findings reported in a systematic review by Spronk et al. The study team reviewed DSIs explicitly developed for use among patients diagnosed with colorectal cancer and lung cancer [21]. In one report, Dubenske et al. described an interactive web-based communication system for patients with advanced lung cancer and family members that provided patients access to information about treatment alternatives, enabled patients to clarify personal treatment preference, and generated a summary report for clinicians. They found that patients guided through this DSI reported a more significant reduction in symptom distress than controls [22]. In another report, Meropole et al. tested a web-based DSI designed to help patients with advanced cancer clarify treatment preference and train patients to discuss treatment options with their provider. Following clinical consultation, patients exposed to this type of DSI reported greater satisfaction with physician communication and treatment decision-making than controls [23].

Our study found that using the DSI prototype was feasible, as it required ~15 min in the waiting room or while the patient was waiting for their oncologist consultation. As described above, we also determined that deployment of the DSI prototype had beneficial effects on knowledge and perceptions related to treatment decision making in a subset of patients included in the pilot study.

Integrating DSI use in routine care will involve eliciting recommended treatment options from clinicians before the patient visit and training nurses and other allied staff in DSI use. Oncologists in our study were amenable to identifying treatment alternatives they planned to discuss with their patients, and oncology nurses were enthusiastic about the opportunity to engage patients in a structured review of these options. We believe that the importance of nurse-patient interactions that facilitate patient education in cancer care will grow [24]. In this regard, team care in oncology will be critical in addressing gaps in patient and family member needs. Moreover, team-based care can also help facilitate access to information and engagement in decision-making about initial treatment and subsequent treatment, rehabilitation, and survivorship care [25,26,27].

Regarding study limitations, we developed and piloted the implementation of a DSI in only one oncology practice setting. Additionally, we did not observe the physician-patient interaction, which led to the 30-day treatment status of participants. In addition, the pilot study included only a small number of patients in the study, and, as a result, we could not conduct statistical analyses of DSI effects. Furthermore, the DSI included information about the range of treatment options. Given the dynamic nature of treatment research, it is clear that care plan cards included in the DSI will need to be updated following changes in the evolution of treatment guidelines. It is essential to point out that randomized studies involving more significant numbers of patients and conducted in various practice settings are needed. Such efforts should include adapting and developing care plan option cards that provide patients with basic information about treatment options that evolve in personalized care. Moreover, research is needed to illuminate interactions that take place between a well-informed patient who has expressed a treatment preference and clinicians who embrace shared decision making.

## 5. Conclusions

This project aimed to develop a novel, patient-friendly DSI that can be integrated into routine care. The DSI described here was implemented in real-time in the clinic through collaboration with oncology nurses. We showed it helped patients with newly diagnosed advanced NSCLC prepare for treatment decision-making in the physician-patient encounter. Further research is needed to evaluate DSI implementation in more extensive and more diverse patient populations and different practice settings. Such studies should be designed to illuminate intervention effects on patient treatment knowledge, decisional conflict, preference, and choice.

## Figures and Tables

**Table 1:** Themes identified in interviews with physician and patient panel members.

Physician Panel Themes	Patient Panel Themes
Tailoring Treatment to the patient	Patient and caregiver involvement
Physician-patient communication	Physician-patient communication
Engaging members of the clinical team	Engaging members of the clinical team
Integrating DSIs in clinical care	Integrating DSIs in clinical care

**Table 2:** Participant background characteristics (*N* = 5).

Characteristic	Category	*N*	%
Sex	Female	3	60.0
	Male	2	40.0
Race	Black or African American	1	20.0
	White	4	80.0
Ethnicity	Not Hispanic or Latino	5	100.0
Marital Status	Married	2	40.0
	Single	2	40.0
	Widowed	1	20.0
Education	Less than a High School Diploma	0	0.0
	High School Diploma	2	40.0
	Some College or Associate Degree	2	40.0
	Bachelor’s Degree	1	20.0
	Master’s Degree or higher	0	0.0

**Table 3:** Change in perceptions about treatment (*N* = 5).

Variable		Baseline Survey	Exit Survey
Belief in Cure From Treatment	Yes	2	2
	No	1	2
	Unsure	2	1
Awareness of Treatment Options	Yes	2	5
	No	1	0
	Unsure	2	0
Concern about Treatment Costs	Yes	3	1
	No	2	3
	Unsure	0	1

**Table 4:** Participant certainty in treatment decision making (*N* = 5).

Variable	Mean Baseline Survey Score	Mean Exit Survey Score	Change
Decisional Conflict Scale	2.2	3.2	+1.0

A mean scale score of <3 indicates a low level of certainty in decision making, while a mean score of >3 indicates a high level of certainty in decision making. A positive change score indicates increased certainty.

**Table 5:** Recommended treatment options, preference, and status at 30 days.

Participant	Treatment Option 1	Treatment Option 2	Treatment Preference	Treatment Status
1	Chemotherapy and Immunotherapy	Immunotherapy	Chemotherapy and Immunotherapy	Undecided
2	Chemotherapy and Immunotherapy	Chemotherapy	Chemotherapy and Immunotherapy	Chemotherapy and Immunotherapy
3	Immunotherapy	Clinical Trial	Immunotherapy	Clinical Trial
4	Chemotherapy	Clinical Trial	Chemotherapy	Chemotherapy
5	Immunotherapy	Supportive Care	Immunotherapy	Chemotherapy

## Supplementary Materials

The following are available online at https://www.mdpi.com/article/10.3390/jpm11100998/s1: ST1. Studies of Treatment Decision Support Interventions among Patients Diagnosed with Late-Stage Non-Small Cell Lung Cancer, Care Plan Option Preference Report, Care Plan Card, Chemotherapy and Immunotherapy.
