# Supplementary material for: Engaging Patients with Late-Stage Non-Small Cell Lung Cancer in Shared Decision Making about Treatment
Source: J Pers Med. 2021 Oct 1;11(10):998. doi: 10.3390/jpm11100998 (PMC8539978; doi:10.3390/jpm11100998)
Supplement: Supplementary file 1 [file jpm-11-00998-s001.zip › Care-Plans-Cards updated 9-30-2021.pdf]

Care Plan Card: Chemotherapy and Immunotherapy

**Common Questions:**	**Chemotherapy and Immunotherapy**
Can treatment cure my disease?	Research indicates that there is no cure at this time. Treatment is intended to increase length and quality of life.
What will I receive?	1-2 chemotherapy drugs along with an immunotherapy drug.<br>Supportive care that is focused on providing patients relief from physical, emotional, spiritual and financial problems associated with their cancer.
Will my symptoms be treated?	Yes, medications to relieve symptoms may be prescribed.
Will I be offered radiation or minor surgery if it could relieve my symptoms?	Yes
How often do I get treatment?	Usually every 3 weeks. Treatments are given intravenously.
How often will I need x-rays or CAT scans?	Usually every 9 weeks, but the schedule may vary.
How often will I need blood tests?	1-2 visits every 3 weeks or more frequently if symptoms occur.
What are the side effects?	Chemotherapy: Side effects may vary based on the drug (dehydration, muscle weakness, fever, fatigue, nausea, and rarely hair loss).<br><br>Immunotherapy: Mild fatigue in most cases. More serious side effects can occur (loss of thyroid function and liver irritation), but are rare.
What is the out-of-pocket cost of treatment?	Given your current insurance coverage, the estimated out of pocket maximum cost for treatment will be $_______.Our financial advocates are available to help you to manage this cost.

Care Plan Card 2: Immunotherapy

Common Questions:	Immunotherapy
Can treatment cure my disease?	Research indicates that there is no cure at this time. Treatment is intended to increase length and quality of life.
What will I receive?	Usually one drug is used.<br>Supportive care that is focused on providing patients relief from physical, emotional, spiritual and financial problems associated with their cancer.
Will my symptoms be treated?	Yes, medications to relieve symptoms may be prescribed.
Will I be offered radiation or minor surgery if it could relieve my symptoms?	Yes
How often do I get treatment?	Usually every 3 weeks. Treatments are given intravenously.
How often will I need x-rays or CAT scans?	Usually every 9 weeks, but the schedule may vary.
How often will I need blood tests?	1-2 visits every 3 weeks or more frequently if symptoms occur.
What are the side effects?	Mild fatigue in most cases. More serious side effects can occur (loss of thyroid function and liver irritation), but are rare.
What is the out-of-pocket cost of treatment?	Given your current insurance coverage, the estimated out of pocket maximum cost for treatment will be $_______.Our financial advocates are available to help you to manage this cost.

Care Plan Card 3: Chemotherapy

<u>Common Questions:</u>	<u>Chemotherapy</u>
Can treatment cure my disease?	Research indicates that there is no cure at this time. Treatment is intended to increase length and quality of life.
What will I receive?	1-2 chemotherapy drugs.<br>Supportive care that is focused on providing patients relief from physical, emotional, spiritual and financial problems associated with their cancer.
Will my symptoms be treated?	Yes, medications to relieve symptoms may be prescribed.
Will I be offered radiation or minor surgery if it could relieve my symptoms?	Yes
How often do I get treatment?	Usually 1-2 times every 3 weeks. Treatment is given intravenously.
How often will I need x-rays or CAT scans?	Usually every 9 weeks, but the schedule may vary.
How often will I need blood tests?	1-2 visits every 3 weeks or more frequently if symptoms occur.
What are the side effects?	Chemotherapy: Side effects may vary based on the drug (dehydration, muscle weakness, fever, fatigue, nausea, and rarely hair loss).
What is the out-of-pocket cost of treatment?	Given your current insurance coverage, the estimated out of pocket maximum cost for treatment will be $_______.Our financial advocates are available to help you to [illegible]

Care Plan Card 4: Targeted Therapy

**Common Questions:**	**Targeted Therapy**
Can treatment cure my disease?	Research indicates that there is no cure at this time. Treatment is intended to increase length and quality of life.
What will I receive?	Usually one drug is used.<br>Supportive care that is focused on providing patients relief from physical, emotional, spiritual and financial problems associated with their cancer.
Will my symptoms be treated?	Yes, medications to relieve symptoms may be prescribed.
Will I be offered radiation or minor surgery if it could relieve my symptoms?	Yes
How often do I get treatment?	Once or twice daily orally (pill form).
How often will I need x-rays or CAT scans?	Usually every 3 months.
How often will I need blood tests?	Every 4 weeks or more frequently if symptoms occur.
What are the side effects?	Side effects may vary based on the drug (rash, diarrhea, muscle weakness, fever, fatigue, nausea, and rarely hair loss).
What is the out-of-pocket cost of treatment?	Given your current insurance coverage, the estimated out of pocket maximum cost for treatment will be $_______.Our financial advocates are available to help you to manage this cost.

Care Plan Card 5: Supportive Care

<u>Common Questions:</u>	<u>Supportive Care</u>
Can treatment cure my disease?	Research indicates that there is no cure at this time. Treatment is intended to improve <u>quality</u> of life.
What will I receive?	Supportive care that is focused on providing patients relief from physical, emotional, spiritual and financial problems associated with their cancer.<br><br>No cancer directed drugs. Medication and/or interventions to relive symptoms from cancer will be prescribed.
Will my symptoms be treated?	Yes, medications and/or interventions to relieve symptoms may be prescribed.
Will I be offered radiation or minor surgery if it could relieve my symptoms?	Yes
How often do I get treatment?	There is no cancer specific treatment. Any medication should be taken as prescribed.
How often will I need x-rays or CAT scans?	Depends on symptoms. Hospice option may not include blood tests or scans.
How often will I need blood tests?	When pain or other symptoms occur.
What are the side effects?	Side effects may vary based on medication (constipation, dry mouth, mild nausea).
Is hospice the same as supportive care?	No. Hospice is an insurance benefit that is provided for a person with a terminal illness whose doctor believes he or she has 6 months or less to live if the illness runs its natural course.
What is the out-of-pocket cost of treatment?	Given your current insurance coverage, the estimated out of pocket maximum cost for treatment will be $_______.Our financial advocates are available to help you to manage this cost.

Care Plan Card 6: Clinical Trial

**Common Questions:**	**Clinical Trial**
Can participating in a clinical trial cure my disease?	Clinical trials are research studies that aim to find *new ways* to prevent, treat and manage cancer. Data from patients is collected over time and compared to a different group of people receiving standard care.
What will I receive?	Clinical trials that test *new ways* of treating cancer, such as a new device, drug or procedure.<br>Supportive care that is focused on providing patients relief from physical, emotional, spiritual and financial problems associated with their cancer.
Will my symptoms be treated?	Yes, medications to relieve symptoms may be prescribed. In addition to the potential benefits of living longer, relief of symptoms, remission, and improved quality of life, a clinical trial provides access to a new treatment that may be more effective than standard care.
Will I be offered medications or procedures if they could relieve my symptoms?	Yes
How often do I get treatment?	Treatment would be administered according to trial requirements.
How often will I need blood tests, x-rays, or CAT scans?	Procedures would be performed according to trial requirements.
What are the side effects?	New treatments may have side effects that are not experienced in standard care. More visits to the doctor and extra tests may be required.
How can I learn more about the clinical trial I am eligible for?	Your doctor can answer many of your questions. You can also learn more at:<br>Jefferson 215-955-8874 #4 (between the hours of 8:00 a.m. - 4:45 p.m.)<br>• https://www.cancer.gov/about-cancer/treatment/clinical-trials/what-are-trials/types<br>• https://clinicaltrials.gov/<br>• http://www.kimmelcancercenter.org/cancer-center.html
What is the out-of-pocket cost of treatment?	Given your current insurance coverage, the estimated out of pocket maximum cost for treatment will be $_______.Our financial advocates are available to help you to [illegible]
