# Supplementary material for: Engaging Patients with Late-Stage Non-Small Cell Lung Cancer in Shared Decision Making about Treatment
Source: J Pers Med. 2021 Oct 1;11(10):998. doi: 10.3390/jpm11100998 (PMC8539978; doi:10.3390/jpm11100998)
Supplement: Supplementary file 1 [file jpm-11-00998-s001.zip › jpm-1320437 ST1.pdf]

Supplementary Table S1 (ST1). Studies of Treatment Decision Support Interventions among Patients Diagnosed with Late-Stage Non-Small Cell Lung Cancer					
**Decision Support Interventions**	**First Author (year)**	**Study Population**	**Design**	**Outcome Measures**	**Outcomes**
Booklet with information on lung cancer, staging, functional impact of the disease and coping strategies, treatment options of radiation therapy and chemotherapy plus supportive care chemotherapy benefits and risks, treatment decision making<br>audio recording of booklet content, and worksheet to clarify values and treatment preference	Fiset et al., (2000)	Patients (N=20) with Stage IV NSCLC attending outpatient clinics at cancer centers	Uncontrolled observational study of patient pre- and post-intervention outcomes	Pre-post change in patient knowledge, perceptions of treatment options, treatment decisional conflict, and intervention acceptability	Knowledge about treatment options, benefits, and risks increased ($p < 0.001$) after exposure to the intervention<br>Favorable perceptions of chemotherapy increased ($p = 0.013$)<br>Treatment decisional conflict decreased ($p < 0.001$)<br>Intervention provided information that was about the right length (75%), clear (75%), helpful (100%), and balanced (75%)
Booklet with information on lung cancer diagnosis and prognosis, chemotherapy and supportive care, treatment benefits and risks, survival, quality of life effects, and a values clarification exercise<br>audio recording of booklet content	Leighl et al., (2006)	Patients (N=20) with Stage IV NSCLC attending outpatient clinics at cancer centers in Canada	Uncontrolled observational study of patient pre- and post-intervention outcomes	Pre-post change in patient knowledge and anxiety, and<br>post-intervention acceptability	Knowledge about chemotherapy and supportive care increased ($p < 0.001$) after exposure to the intervention<br>Anxiety decreased ($p = 0.04$)<br>Intervention provided information that was about the right amount (85%), about the right length (60%), clear (95%), very helpful (100%), very appropriate (80%), and very useful (80%), and balanced (80%)
