# Supplementary material for: Engaging Patients with Late-Stage Non-Small Cell Lung Cancer in Shared Decision Making about Treatment
Source: J Pers Med. 2021 Oct 1;11(10):998. doi: 10.3390/jpm11100998 (PMC8539978; doi:10.3390/jpm11100998)
Supplement: Supplementary file 1 [file jpm-11-00998-s001.zip › Preference-Report updated 9-30-2021.pdf]

Care Plan Option Preference Report

PATIENT: **NURSE:**

DOB: **CONTACT INFO:** **ADDRESS:**

APPOINTMENT DATE: ____/____/____ **APPOINTMENT TIME:**

	CARE PLAN OPTIONS	
**PREFERENCE**	**Option __**	**Option __**
**Do Not Prefer**	☐	☐
**Unsure**	☐	☐
**Prefer**	☐	☐

Care Plan Option 1: Chemotherapy and Immunotherapy
Care Plan Option 2: Immunotherapy
Care Plan Option 3: Chemotherapy
Care Plan Option 4: Targeted Therapy
Care Plan Option 5: Supportive Care
Care Plan Option 6: Clinical Trial

Nurse Comments:
